# Clinical and Molecular Heterogeneity of RTEL1 Deficiency

**DOI:** 10.3389/fimmu.2017.00449

**Published:** 2017-05-01

**Authors:** Carsten Speckmann, Sushree Sangita Sahoo, Marta Rizzi, Shinsuke Hirabayashi, Axel Karow, Nina Kathrin Serwas, Marc Hoemberg, Natalja Damatova, Detlev Schindler, Jean-Baptiste Vannier, Simon J. Boulton, Ulrich Pannicke, Gudrun Göhring, Kathrin Thomay, J. J. Verdu-Amoros, Holger Hauch, Wilhelm Woessmann, Gabriele Escherich, Eckart Laack, Liliana Rindle, Maximilian Seidl, Anne Rensing-Ehl, Ekkehart Lausch, Christine Jandrasits, Brigitte Strahm, Klaus Schwarz, Stephan R. Ehl, Charlotte Niemeyer, Kaan Boztug, Marcin W. Wlodarski

**Affiliations:** ^1^Department of Pediatrics and Adolescent Medicine, Division of Pediatric Hematology and Oncology, Medical Centre, Faculty of Medicine, University of Freiburg, Freiburg, Germany; ^2^Center for Chronic Immunodeficiency, Medical Center, Faculty of Medicine, University of Freiburg, Freiburg, Germany; ^3^Spemann Graduate School of Biology and Medicine, University of Freiburg, Freiburg, Germany; ^4^Department of Rheumatology and Clinical Immunology, Medical Centre, Faculty of Medicine, University of Freiburg, Freiburg, Germany; ^5^Department of Paediatrics, Univeristy of Bern, Bern, Switzerland; ^6^Ludwig Boltzmann Institute for Rare and Undiagnosed Diseases, Vienna, Austria; ^7^CeMM Research Center for Molecular Medicine of the Austrian Academy of Sciences, Vienna, Austria; ^8^Department of Pediatric Hematology and Oncology, Children’s Hospital, University of Cologne, Cologne, Germany; ^9^Department of Medical Genetics, Biozentrum, University of Wuerzburg, Wuerzburg, Germany; ^10^Telomere Replication and Stability Group, MRC London Institute of Medical Sciences (LMS), London, UK; ^11^Institute for Transfusion Medicine, Institute for Clinical Transfusion Medicine and Immunogenetics Ulm, German Red Cross Blood Service Baden-Wuerttemberg – Hessen, University Ulm, Ulm, Germany; ^12^Department of Human Genetics, Hannover Medical School, Hannover, Germany; ^13^Department of Pediatric Hematology and Oncology, Justus-Liebig-University, Giessen, Germany; ^14^Clinic of Pediatric Hematology and Oncology, University Medical Center Hamburg-Eppendorf, Hamburg, Germany; ^15^Hemato-Oncology Clinic Hamburg, Hamburg, Germany; ^16^Faculty of Medicine, Institute of Pathology, Medical Center, University of Freiburg, Freiburg, Germany; ^17^German Cancer Consortium (DKTK), Freiburg, Germany; ^18^German Cancer Research Center (DKFZ), Heidelberg, Germany; ^19^Department of Pediatrics and Adolescent Medicine, Medical University of Vienna, Vienna, Austria; ^20^St. Anna Kinderspital and Children’s Cancer Research Instutute, Department of Pediatrics and Adolescent Medicine, Medical University of Vienna, Vienna, Austria

**Keywords:** RTEL1, dyskeratosis congenita, bone marrow failure, immunodeficiency, lymphopenia

## Abstract

Typical features of dyskeratosis congenita (DC) resulting from excessive telomere shortening include bone marrow failure (BMF), mucosal fragility, and pulmonary or liver fibrosis. In more severe cases, immune deficiency and recurring infections can add to disease severity. RTEL1 deficiency has recently been described as a major genetic etiology, but the molecular basis and clinical consequences of RTEL1-associated DC are incompletely characterized. We report our observations in a cohort of six patients: five with novel biallelic *RTEL1* mutations p.Trp456Cys, p.Ile425Thr, p.Cys1244ProfsX17, p.Pro884_Gln885ins53X13, and one with novel heterozygous mutation p.Val796AlafsX4. The most unifying features were hypocellular BMF in 6/6 and B-/NK-cell lymphopenia in 5/6 patients. In addition, three patients with homozygous mutations p.Trp456Cys or p.Ile425Thr also suffered from immunodeficiency, cerebellar hypoplasia, and enteropathy, consistent with Hoyeraal-Hreidarsson syndrome. Chromosomal breakage resembling a homologous recombination defect was detected in patient-derived fibroblasts but not in hematopoietic compartment. Notably, in both cellular compartments, differential expression of 1243aa and 1219/1300aa RTEL1 isoforms was observed. In fibroblasts, response to ionizing irradiation and non-homologous end joining were not impaired. Telomeric circles did not accumulate in patient-derived primary cells and lymphoblastoid cell lines, implying alternative pathomechanisms for telomeric loss. Overall, RTEL1-deficient cells exhibited a phenotype of replicative exhaustion, spontaneous apoptosis and senescence. Specifically, CD34^+^ cells failed to expand *in vitro*, B-cell development was compromised, and T-cells did not proliferate in long-term culture. Finally, we report on the natural history and outcome of our patients. While two patients died from infections, hematopoietic stem cell transplantation (HSCT) resulted in sustained engraftment in two patients. Whether chemotherapy negatively impacts on the course and onset of other DC-related symptoms remains open at present. Early-onset lung disease occurred in one of our patients after HSCT. In conclusion, RTEL deficiency can show a heterogeneous clinical picture ranging from mild hypocellular BMF with B/NK cell lymphopenia to early-onset, very severe, and rapidly progressing cellular deficiency.

## Introduction

Dyskeratosis congenita (DC) comprises a group of Mendelian disorders marked by intrinsic telomere shortening, caused by defects in genes involved in telomere homeostasis. The varied clinical phenotype and delayed onset of non-hematological symptoms can render the diagnosis of DC difficult. The classical form of DC is characterized by bone marrow failure (BMF), mucocutaneous features, and pulmonary and/or hepatic fibrosis (1–4). In patients with severe and early-onset disease, also referred to as Hoyeraal-Hreidarsson syndrome (HHS), in addition to BMF, the disease is distinguished by intrauterine growth retardation, microcephaly/cerebellar hypoplasia, and increased susceptibility to infections due to cellular and humoral immunodeficiency (5–7). The most consistent immunological phenotype is a combined decrease in B- and NK-cells with usually normal numbers and *in vitro* function of T-cells (8).

Until recently, HHS has been associated with mutations in *TINF2* (heterozygous), *DKC1* (X-linked), or *TERT* (homozygous) but its cause remains elusive in approximately half of the patients (1). Ballew et al. and Walne et al. first reported the identification of biallelic *RTEL1* mutations in patients with HHS (9, 10). To date, 30 distinct mutations have been reported in 24 unrelated pedigrees, in patients suffering from similar clinical features including BMF, B-/NK-cell lymphopenia, and developmental delay (9–20). In addition, heterozygous missense variants in *RTEL1* have been identified in association with idiopathic pulmonary fibrosis, reported in 10 pedigrees (21–23). RTEL1 is a helicase essential in DNA metabolism (24–27) and has been classified as a helicase with a conserved iron–sulfur (FeS) cluster. Other disorders resulting from mutations in FeS-helicase genes include Xeroderma pigmentosum (*XPD*), Warsaw breakage syndrome (*DDX11/ChIR1*), and Fanconi anemia group J (*FANCJ*). Despite similar pathophysiological basis, the clinical and biological phenotypes are different in these entities (25, 28). Inappropriate resolution of the telomeric-loops (T-loops) into free telomeric circles (T-circles) has been postulated as the mechanism underlying catastrophic telomere shortening and cellular defect in RTEL1 deficiency (10). However, due to the limited experimental studies in patients, the molecular basis of the clinical phenotype has remained incompletely characterized.

Here, we examined the natural history and treatment outcome of six patients with five novel *RTEL1* mutations. To better understand the functional consequences of identified mutations, we employed molecular and cellular assays in patient-derived primary cells, long-term culture, and manipulated cell lines. We ascribe a premature truncation effect on mRNA level to the splice site mutation c.2652 + 5G>A. We also demonstrate normal V(D)J recombination and unaffected T-loop disassembly with normal numbers of T-circles in RTEL1-deficient patients, extending previous findings in RTEL1 deficiency. Our clinical and experimental observations support the notion of early proliferative exhaustion along with spontaneous apoptosis, increased senescence, and rapid telomere shortening in *RTEL1*-mutated cells. Moreover, we report on the clinical course of hematopoietic stem cell transplantation (HSCT) in two of our patients.

## Materials and Methods

### Telomere Length Assessment and Genetic Studies

For initial explorative analysis, the relative telomere length (RTL) was measured from DNA of granulocytes using quantitative multiplex real-time polymerase chain reaction (PCR) according to Cawthon (29). In-house reference values from peripheral blood (PB) and bone marrow (BM) granulocytes of 90 healthy BM donors (age 2–18 years) were used for percentile calculation. RTL [telomere to single copy gene (T/S) ratio] was calculated as median from at least two independent triplicate runs. The intra-assay coefficient of variation (CV) ranged from 0 to 6% (mean 3.75%), and inter-assay CV was 2–11% (mean 7%). Telomere length was validated using metaphase telomere/centromere-fluorescence *in situ* hybridization (T/C-FISH) in a second laboratory, as previously described (30).

### Cell Culture and Immunological Studies

Primary fibroblasts were grown in DMEM medium containing 20% FCS and 1% P/S and tested mycoplasma free. For hematopoietic cell cultures, mononuclear cells (MNCs) were isolated from BM of patients and healthy control using Ficoll-based density gradient centrifugation, and magnetically isolated CD34^+^ cells (MACS Miltenyi) were cultured for 7 days with IL6, SCF, and FLT3-L. T-cell proliferation, flow cytometric analysis of lymphocyte subsets, T-cell receptor Vβ-repertoire, and T-cell cytokine production were assessed as previously reported (31, 32). For analysis of *ex vivo* survival, MNCs were cultured for 6 days. At days 0, 1, 2, 3, and 6, 2 × 10^5^ cells were stained with Annexin V (AV) and PI (BD Biosciences) and analyzed by flow cytometry. Degranulation of T- and NK-cells was assessed as previously reported (33).

### Senescence-Associated β-Galactosidase Staining

Primary fibroblasts from healthy control, P1, and a patient with known DC and *TERC* mutation were fixed for 5 min in 2% vol/vol paraformaldehyde in PBS, washed in PBS, and stained in β-galactosidase fixative solution (X-gal) in 5 mmol/l potassium ferricyanide, 5 mmol/l potassium ferrocyanide, and 2 mmol/l MgCl_2_ in PBS for 16 h at 37°C. Controls and patient cells were analyzed at the same passage number, 200 cells were counted per well, and staining performed in triplicates.

### Radiosensitivity and Mitomycin C (MMC)-Induced Chromosomal Breakage

Primary fibroblasts were seeded at a density of 4,000 cells/cm^2^. Parallel cultures were grown in DMEM with GlutaMAX (Gibco) and supplemented with 15% FBS (PAN). For flow cytometry, 48 h cultures were left untreated or exposed to 10 ng/ml MMC (Medac) or initially irradiated with 1.5 Gy from a linear accelerator. Cells were detached using 1× (0.05%) trypsin (diluted from trypsin 0.5%-EDTA 0.2% solution 10×, PAA), pelleted, and stained in medium containing 15 µg/ml Hoechst dye 33342 (Molecular Probes) for 30 min in the dark. Gates were set on vital cells via propidium iodide (PI, 1 µg/ml) exclusion. Split samples were stained with 1 µg/ml 4′,6-diamidino-2-phenylindole (DAPI; Molecular Probes) in buffer containing 154 mM NaCl, 0.1 M Tris pH 7.4, 1 mM CaCl_2_, 0.5 mM MgCl_2_, 0.2% BSA, and 0.1% NP40 in dH_2_O. Univariate flow histograms were recorded on a triple-laser equipped LSRII flow cytometer (Becton Dickinson) using UV excitation of Hoechst 33342 or DAPI, and 488-nm excitation of PI. Resulting cell cycle distributions reflecting cellular DNA content were quantified using the MPLUS AV software package (Phoenix Flow Systems).

### Cytogenetic Analyses for Visualization of Chromosomal Breakage

Fibroblasts were exposed to MMC at final concentrations of 0, 10, 50, or 100 ng/ml for the final 24 h of culture. For the last 3 h, 16 µl of Colcemid Solution (10 µg/ml; PAA) per milliliter of growth medium were added. Metaphase preparation followed standard procedures. Detachment of cells was evaluated by trypsin as above. Pellets were subjected to hypotonic treatment using 10 ml of pre-warmed 0.075 M KCl for 10 min at 37°C. Nuclei and metaphases were fixed using freshly prepared, ice-cold 100% methanol, and glacial acetic acid 3:1. A minimum of 50 complete metaphases from Giemsa-stained slides of each MMC concentration were scored quantitatively for breakage rates and analyzed qualitatively for types of chromosome aberrations.

### V(D)J Recombination Assay

V(D)J recombination assays were performed as described previously (34). In short, human primary RTEL1-proficient or RTEL1-deficient dermal fibroblasts were transfected using the Cell Line V Nucleofector Kit (Lonza, Cologne, Germany). Transfections were performed with 1.2 µg pcWT-RAG1, 1.8 µg pcWT-RAG2, 8.0 µg pMACS11-19VDJ, or pMACS11-19Flip, and 1.0 µg pcDNA6/myc-His Version A (Invitrogen, Life Technologies, Darmstadt, Germany). After 48 h, cells were harvested and analyzed by immunofluorescence flow cytometry. Transfected fibroblasts were detected with biotin–anti mouse H-2Kk antibody (BD Biosciences, San Jose, CA, USA) against the truncated murine major histocompatibility complex class I protein H-2Kk additionally encoded by the pMACS11-19VDJ and pMACS11-19Flip plasmids. The biotin-labeled antibodies were detected by streptavidin–peridinin chlorophyll protein staining (BD Biosciences). Assays were performed three times independently for the two tested cell lines. The arithmetic means of the three values and the corresponding SDs were calculated.

### T-Circle Analysis

The analysis of telomeric T-circles was performed as previously described (27) using patient fibroblasts (P1, P3), T-lymphoblasts (P1 and family members, P5), and cell lines [VA13 ALT, *Rtel1^−/−^* mouse embryonic fibroblasts (MEFs), *Rtel1*^F/F^ MEFs, and BJhTERT] and as positive control fibroblasts with homozygous mutation p.Arg1264His known to exhibit T-circle loss.

### Genomic Studies

#### Exome Sequencing (ES) Studies and Protein Modeling

For homozygosity mapping in P1 and P2, homozygous regions were mapped as described before (35). In brief, genomic DNA was treated according to the Affymetrix^®^ Genome-Wide Human SNP Nsp/Sty 6.0 protocol. Results were assessed using the Affymetrix^®^ Genotyping Console™ software, PLINK (36).

##### ES in P1 and P2

The sample was prepared using the Illumina TruSeq DNA Sample Preparation Guide, the Illumina TruSeq Exome Enrichment Guide version 3, and the TruSeq PE Cluster Kit v3 Reagent Preparation Guide. Data were analyzed by applying Burrows–Wheeler Aligner for the alignment of the reads and the Genome Analysis Toolkit (37, 38) for quality score recalibration as previously described (35). Annotation was done using the ANNOVAR (39).

##### Variant Validation

The variants of the final hit list were validated with capillary sequencing on genomic DNA from the patients, using Big Dye Terminator v3.1 Cycle Sequencing Kit (Applied Biosystems, Germany) on a 3130xl Genetic Analyzer (Applied Biosystems).

##### Protein Modeling and Phylogenetic Conservation

The protein model (AA 1–754) was created using I-TASSER (39) based on the crystal structure of the protein database molecule 2vsfA (40) (sequence homology 20%). Secondary structures were assigned with the program ICM-Browser, Molsoft LLC. Phylogenetic conversation was assessed using Polyphen-2.

#### Targeted Sequencing Using Sanger Sequencing

DNA was extracted using Gentra Puregene DNA kit (Qiagen) from PB. PCR and cycle sequencing products were purified using standard enzymatic or sephadex-based cleanup. PCR and Sanger sequencing were performed as previously described (41).

Primers used for amplification and sequencing of genomic DNA:

**Table:** 

Exon	Forward	Reverse	MgCl_2_ (mM)
2	CAGTGCACATGCTCGCATC	ATGACAGACGCTGCCTCTG	2.2
3	GCCTCTGCATCTGCAAAGAG	CTTGAGTTCTGCTTGAGAGAC	2.2
4	TGTCAGATTCTTGGCTGTCTG	AGCGCTCTGCACACTTCG	2.2
5–6	TCCTCCCTCTGTCCAGTAC	CAAGCACAACCAGGCTGTG	1.5
7	CCTCAGTGGGTGCTTTGTG	ACTCTATCTTCCTCAGAGCTG	2.2
8	CAGGATGAGGGCTCCTTC	CCCAGTGACAGAGGTGAG	1.5
9	CTCATCTGCGCTTGTGATGT	CACCTAGGGCTTCAGGAG	2.2
10	GAACTTGGCTGTCAGCCTC	CCCAAGAAGCCTCTGAGAG	2.2
11	GAATCCTGGTTCTCAAGGG	GTCTCCAGGCAGCTCAAC	1.5
12	AGACATTGCAAAGCTGAAGAG	GATGTGACAGCCCAGGAC	1.5
13	ACTTCCACAGTTGTTGCCTTC	GCTGGCAAGTGGCACTAAC	1.5
14	GAGATGGAGCTTGGCAGTC	CTGGAAAGGAGCCGAGAG	2.2
15–16	AGAAAGGGTCAGGCAGGTG	TGAGGAGAATGCTCTGGATTG	2.2
17–18	AAGCTGGCAGGCTCACAC	CACTCACCCAGAGCCTTC	1.5
19–21	TGGAGAACCCACACATCATC	AGGAGCCCTGAAGAGGCA	1.5
22	CTTCATCTTGGAGCATGAGAC	GCCACCTCCAACTCTTGTG	1.5
23–24	CCACAGATGGAGCTTCCTC	GCTTCAAGGGTTCAGTTCAC	2.2
25–26	CTCTCCTTCCCCACATGAG	GCACAAAGCCAGGTGAGTC	2.2
27	AACGCCCCAGGCAAGGAT	AGGACCCACAGACAGCCA	2.2
28	AAGTTGTGGCACTGTCACC	GGCTGTGTCCCTCACATG	2.2
29–30	CCAGTTTCTCAGGCAGCAG	CTCCCATAGGGGAACAGAG	2.2
31	AGGCTGGTGTCTCCTCTGA	AGCAGTCCCCACCATGAGA	1.5
32	GGCTTCACGAGGCTAACTC	CTTTGCTGCTCACTCCCAG	1.5
33–34	CAACTCTTGGCAGCGCTGA	TGAAGGTGCCGTTGCCAG	2.2
34–35	CTCCTGTGCTTACCCACAG	CTATTCTGTTGGGTGGGTTC	2.2
36	AGGTGGCATGTCGGTCAG	TTGTGGGTGGCGTGGCAA	2.2

#### RT-PCR

RNA was prepared according to standard methods (TRIZOL, Invitrogen Lifetech, USA). cDNA was generated from 1 µg of RNA using the QuantiTect Reverse Transcription Kit (Qiagen, Germany). Reverse transcriptase was not added in RT minus (RT^−^) reactions, used for exclusion of genomic DNA contamination and thus unspecific amplification of genomic DNA in the RT-PCR. The PCR master mix was set up as follows: 6 µl of 5× PCR buffer, 3 µl of 2mM deoxynucleoside triphosphates, 1 µl of 10µM each forward and reverse primers, 0.2 µl of Taq (5 U/μl; Go Taq DNA Polymerase, Promega), MgCl_2_ (final 2.2 mM), and 2 µl of cDNA were mixed with sterile water to a final volume of 30 µl. Thermocycling was performed on a Peqstar Thermocycler (Peqlab, Germany). After an initial denaturation step at 94°C for 2 min and 17 touch-down cycles (denaturation at 94°C for 30 s; annealing at 63°C, −0.5°C per step, for 30 s; and extension at 72°C for 30 s), an additional 20 PCR cycles were performed (30 s at 94°C, 30 s at 55°C, and 30 s at 72°C).

RTEL1 cDNA primers:

**Table:** 

Ex3–4 fw	AGACCCCATAGCTTGCTACA	Ex6–7 rev	TCTGTAGATGGTTACTCTCTTG
Ex26–27 fw	AGGTCCTCAGGGTCACCAG	Ex30–31 rev	GTTCTTCCAGTGGGGTCCAG
Ex27–28 fw	CGAGGAGCAGGCCCACAG	Ex29–30 rev	TGGTAGAAGCCTTGGAGCAG
Ex34–35 fw	GCACCTTCAGGCCTCTAG	Ex36 rev	GACGTTGCAGTAGCGGCA

## Results

### Clinical Phenotype and Natural History of Patients with *RTEL1* Mutations

**Figure 1:**
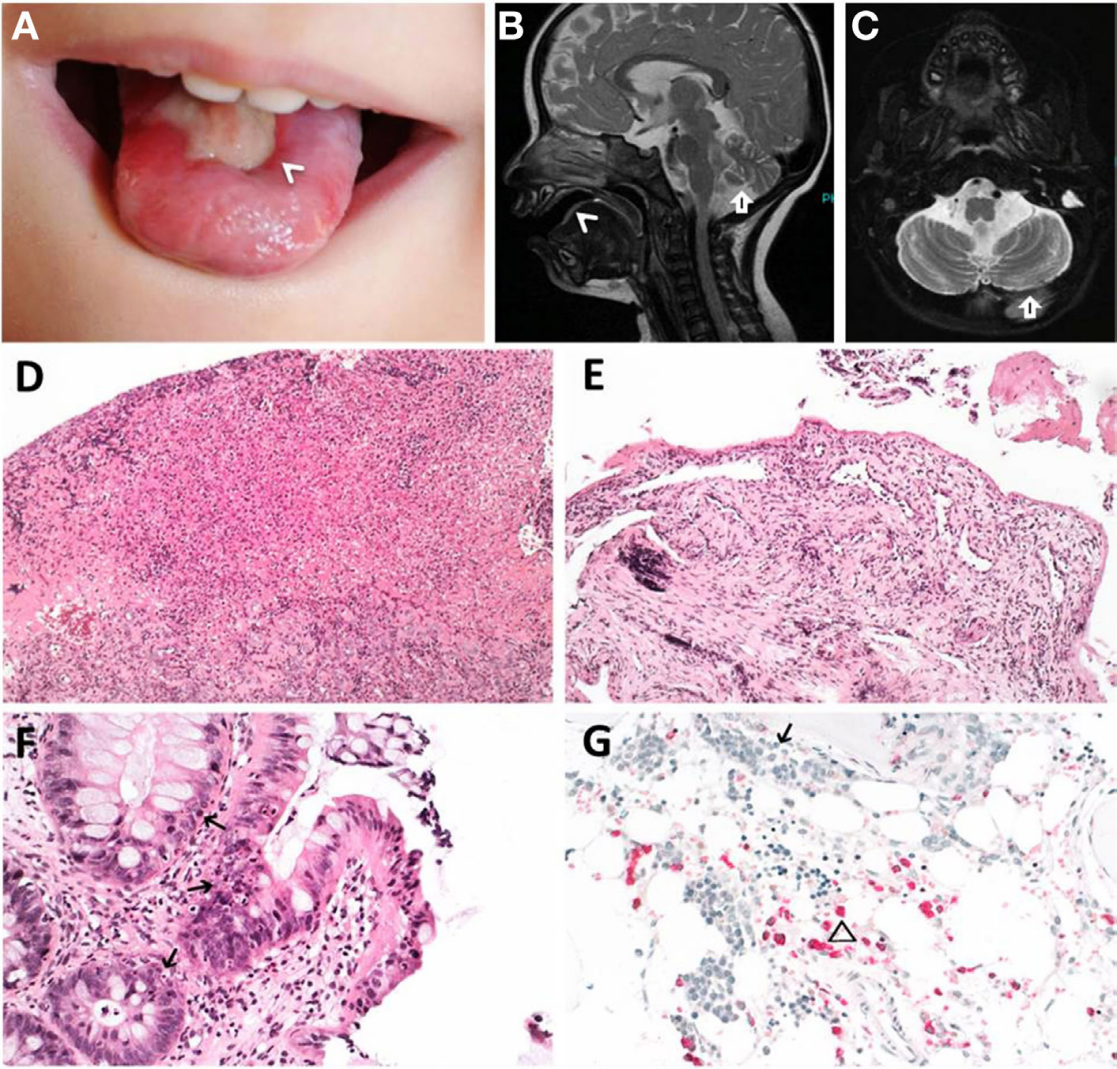
**Clinical features of index patient 1 (P1)**. **(A–C)** Leukoplakia (<), sinistral cerebral hypoplasia (arrow). **(D)** Granulation tissue of tongue and **(E)** esophageal ulcers. **(F)** Apoptotic bodies in colon (arrows). **(G)** Hypocellular bone marrow before hematopoietic stem cell transplantation with immature erythropoiesis (arrow) and sparse myeloid precursors (triangle). **(D–F)** Hematoxylin and eosin staining, **(G)** naphthol-AS-d-chloroacetate esterase (NACE) staining.

**Figure 2:**
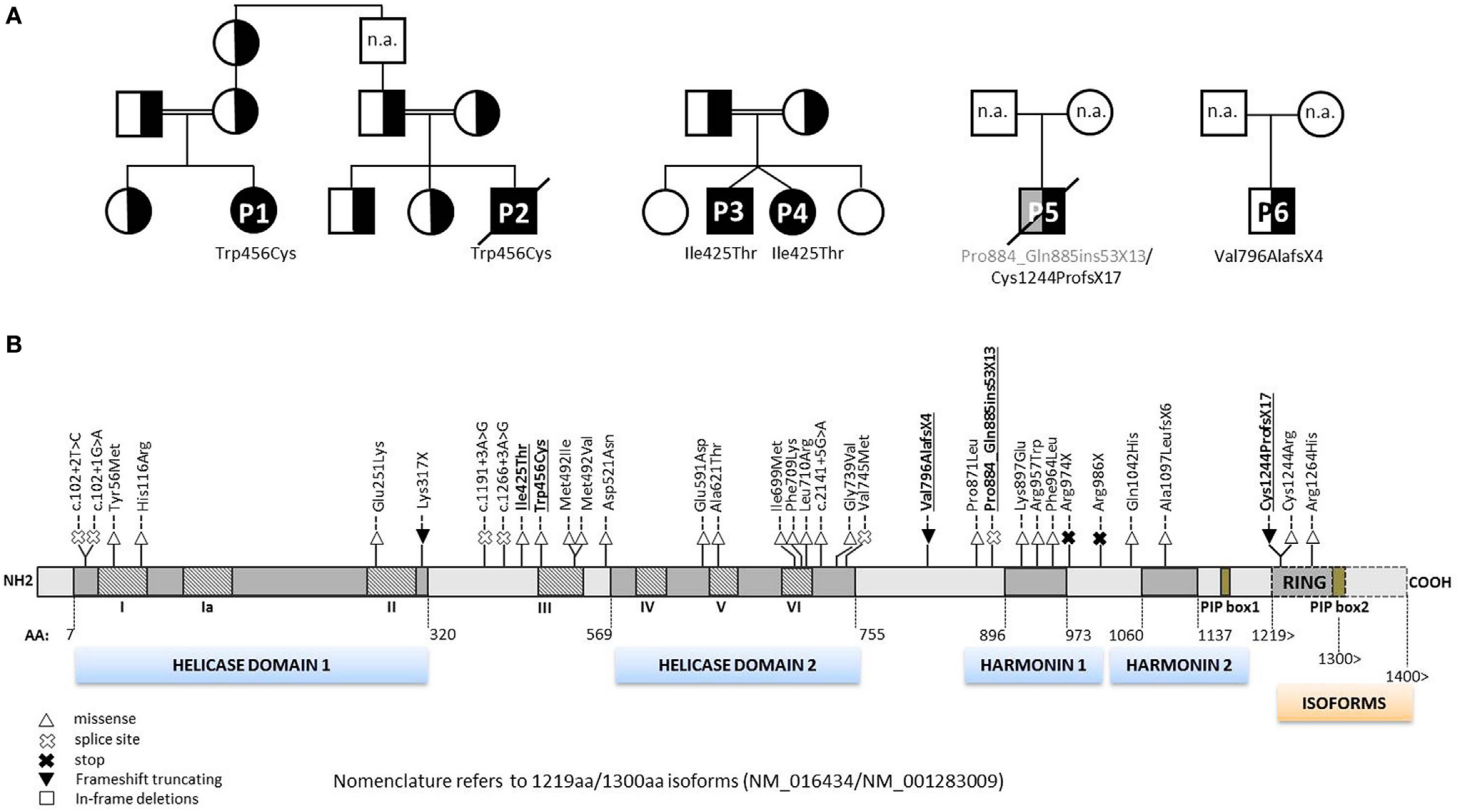
**Pedigrees of RTEL1-deficient patients and annotation of mutations**. **(A)** Six RTEL1-deficient patients with their respective nuclear families are shown. P1 and P2 are part of a large consanguineous pedigree. P3 and P4 are twins. Heterozygous family carriers are indicated by half-filled symbols. P1–P4 carry homozygous missense mutations, P5 and P6 carry biallelic and monoallelic nonsense mutations, respectively. n.a., not analyzed. **(B)** The 1300aa RTEL1 isoform protein structure displaying the novel mutations described in this study (highlighted in bold, underlined font) along with previously reported mutations. Mutations previously reported using 1243aa isoform (NM_032957) in three reports (9, 10, 20) have been adapted to 1300aa isoform (NM_001283009). Heterozygous missense variants identified in association with pulmonary fibrosis (21–23) are not shown. For clarity, mutations are shown on protein level without “p.” as preceding.

The essential clinical and laboratory data of our patients are summarized in Tables 1 and 2, key clinical featured of the index patient are shown in Figure 1, and the pedigrees of the investigated families are shown in Figure 2A.

**Table 1:** **Clinical phenotype of patients 1–6**.

Clinical phenotype	P1	P2	P3	P4	P5	P6
Ethnic origin	Turkish		Moroccan		German	German

Gender	Female	Male	Male	Female	Male	Male

Age/initial presentation at disease onset	9 months/infections	3 months/failure to thrive	12 months/leukoplakia	4 years/diarrhea	9 years/pancytopenia and infections	19 years/thrombocytopenia

Age at last follow-up	7 years 4 months	19 months	7 years 8 months	7 years 8 months	21 years	19 years

Treatment	MFD HSCT	None	Matched unrelated donor HSCT	Careful watching	Oxymetholone	None

Outcome	Alive (HSCT + 4 years 2 months)	Deceased (CMV)	Alive (HSCT 2 years 6 months)	Alive	Deceased (pneumonia)	Alive

*RTEL1* mutations	c.1368G>T; p.Trp456Cys hom		c.1274T>C; p.Ile425Thr hom		c.2652 + 5G>C; p.Pro884_Gln885ins53X13 het and c.3730delTG; p.Cys1244ProfsX17 het	c.2387delT; p.Val796AlafsX4 het

**Major dyskeratosis congenita (DC) features^a^**[Table-fn tfn1]						
Bone marrow failure	+	+	+	−	+	+
Oral leukoplakia	+	+	+	−	+	−
Abnormal skin pigmentation	−	−	−	−	−	−
Nail dystrophy	−	−	−	−	+	−
Telomere length <first percentile	+	+	+	+	+	+

**Other DC-related features**						
Ataxia/cerebellar hypoplasia on MRI	+/+	+/+	+/+	−/n.i.	−/n.i.	−/n.i.
IUGR	+	−	−	−	+	−
Short stature	+	+	+	−	−	−
Microcephaly	+	+	+	−	+	−
Developmental delay	+	+	+	−	−	−
Esophageal stricture or GI ulcerations	+	−	+	−	+	−
Lung fibrosis/liver cirrhosis	+/−	−/−	−/−	−/−	−/+	−/−
Chronic diarrhea	+	+	+	+	+	−
T^+^B^−^NK^−^ immune phenotype	+	+	+	+	+	−

MMC-induced chromosomal breakage	+ fibroblasts	n.i.	+ fibroblasts	n.i.	− blood	n.i.
	− blood		− blood			

Systemic infections	Viral and bacterial		Bacterial	None	Viral and bacterial	none

*^a^As defined in Dokal (1)*.

*hom, homozygous; het, heterozygous; +, present; −, absent; n.i., not investigated; MFD HSCT, matched family donor hematopoietic stem cell transplantation; IUGR, intrauterine growth retardation; MMC, mitomycin C*.

**Table 2:** **Extended laboratory findings of patients with *RTEL1* mutations**.

Laboratory tests	P1	P2	P3	P4	P5	P6
**Blood count, lowest observed values**						
Hemoglobin, g/dl (*N*: 13.5–17.5)	*6.0*	*6.4*	*5.0*	12.4	*6.0*	15.6
Platelets, 10^9^/l (*N*: 150–450)	*23*	*25*	*24*	288	<*10*	*63*
WBC, 10^9^/l (*N*: 4,500–11,000)	*1,100*	*3,500*	*1,020*	*4,400*	*100*	*3,900*
ANC, 10^6^/l (*N*: 1,500–8,000)	*70*	*1,300*	*620*	2,080	*50*	*1,290*
Lymphocytes, 10^6^/l (*N*: 1,000–4,800)	*770*	*2,100*	*290*	1,650	*50*	1,000
MCV >95th percentile	Yes	No	Yes	Yes	Yes	Yes

**T-cells**						
CD3^+^/μl	*724–937* (2,100–6,200)	*970–1,538* (2,100–6,200)	2,016 (700–4,200)	1,480–1,949 (700–4,200)	*140* (700–2,100)	1,254 (700–2,100)
CD4^+^/μl	*457–561* (1,300–3,400)	*790–1,172* (1,300–3,400)	1,258 (300–2,000)	*740–1,011* (300–2,000)	*60* (300–1,400)	818 (300–1,400)
CD8^+^/μl	*251–350* (620–2,000)	*176–357* (620–2,000)	724 (300–1,800)	670–835 (300–1,800)	*70* (200–900)	357 (200–900)
% γ/δ TCR^+^ of CD3^+^	2 (<10)	0.8 (<10)	1.1 (<10)	6.3 (<10)	n.a.	6.4 (<10)
% CD45RA of CD4^+^	71–79 (63–91)	84 (63–91)	66 (53–86)	62 (53–86)	25 (33–66)	51 (33–66)

B-cells CD19^+^/μl	*1–26* (720–2,600)	*2–47* (720–2,600)	*26–74* (200–1,600)	*230–252* (200–1,600)	*2* (100–500)	162 (100–500)

NK cells: CD3^−^D16^+^CD56^+^/μl	*30–63* (180–920)	*4–23* (180–920)	*4–10* (90–900)	*20–50* (90–900)	*20* (90–600)	105 (90–600)

Immunoglobulins (maximal values)						
IgG/IgA/IgM, g/l	<*1/0.1/0.25*	*4.5/0.4/*0.4	13.9/2.3/0.6	9.8/1.1/0.5	*5.3/0.3/*<*0.2*	n.a.
IgE, kU/l	2.1	<2	24.7	24	n.a.	n.a.
Specific IgG^a^	− (EBV, CMV, VZV, tetanus)	+ (rubella, hepatitis B)	+ (EBV, CMV, tetanus, measles, mumps, rubella)	+ (CMV, VZV, PB19, tetanus, measles, mumps, rubella)	− (EBV, CMV, VZV, PB19, measles, mumps, rubella)	n.a.

T-cell proliferation: PHA/anti-CD3	norm/norm	n.a./n.a.	norm/n.a.	n.a./n.a.	norm/n.a.	n.a/n.a.

*^a^All patients were exposed to the indicated antigens either by vaccination or infection and specific IgG were measured prior to i.v. application of immunoglobulins*.

*+, present; −, absent; n.a., not available; norm, normal; WBC, white blood count; ANC, absolute neutrophil count; MCV, mean corpuscular volume; PHA, phytohemagglutinine*.

*Normal age-related values are depicted in parentheses. Italic formatting indicates laboratory values below the age-related normal range*.

Patient P1 (Family 1) was brought to our attention for recurring bacterial and viral pneumonias manifesting from 9 months of age. Pancytopenia, B/NK-cell lymphopenia, and hypogammaglobulinemia were subsequently noted. In short span, the manifestation of leukoplakia and ataxia associated with cerebellar hypoplasia led to the clinical diagnosis of HHS (Figures 1A–C). Between 2 and 3 years of age, P1 developed refractory non-infectious diarrhea and long-segment esophageal narrowing. An increased apoptotic rate within the colon and esophageal mucosa was detected by endoscopy (Figures 1E,F). Due to rapidly progressing BMF (Figure 1G) and immunodeficiency, P1 was transplanted from her HLA-identical grandmother (further details are described below). Similar clinical symptoms were observed in P2, a second-degree cousin of P1 (Figure 2A). Within first 3 months after birth, P2 had failure to thrive and microcephaly, with recurrent infections manifesting from age of 5 months. He died at the age of 19 months due to systemic CMV infection.

Two further patients (P3 and P4) belong to Family 2 (Figure 2A). P3 initially developed leukoplakia at age of 12 months; however, his leading medical concerns were developmental delay and BMF diagnosed at age of 2 years and 9 months. He was successfully transplanted from a 9/10 matched unrelated donor (MUD) at 5.2 years of age. Identical homozygous *RTEL1* mutation was identified in his twin sister (P4) by family screening. P4 initially presented with mild chronic diarrhea, high MCV and NK-cell lymphopenia, and short telomeres at the age of approximately 4 years, but later also developed mild leukoplakia (Tables 1 and 2).

P5 from Family 3 (Figure 2A) suffered from recurrent infections beginning from 6 years of age and progressive BMF after EBV infection at 9 years of age. In contrast to P1–P4, he developed severe mucosal fragility, nail dystrophy, and liver cirrhosis with secondary hypersplenism (Table 1). He became transfusion dependent for RBC and platelets at 15 years of age. Oxymetholone therapy initiated at age of 20 years later resulted in transfusion independency for platelets. However, infectious complications intensified and he died of sepsis after pneumonia at 21 years of age.

P6 from Family 4 (Figure 2A) was born to non-consanguineous parents with an uneventful family history. Postnatal development was normal and he was asymptomatic at diagnosis when he was 17 years old. Complete blood count performed prior to surgery for a shoulder dislocation revealed thrombocytopenia and elevated MCV (Tables 1 and 2). These findings prompted further investigations and a BM biopsy was compatible with the histological diagnosis of hypocellular refractory cytopenia. Telomere analysis and full DC-genetic workup eventually identified RTEL1 deficiency. Family history revealed no malignancies in all patients or their first-degree relatives.

**Figure 3:**
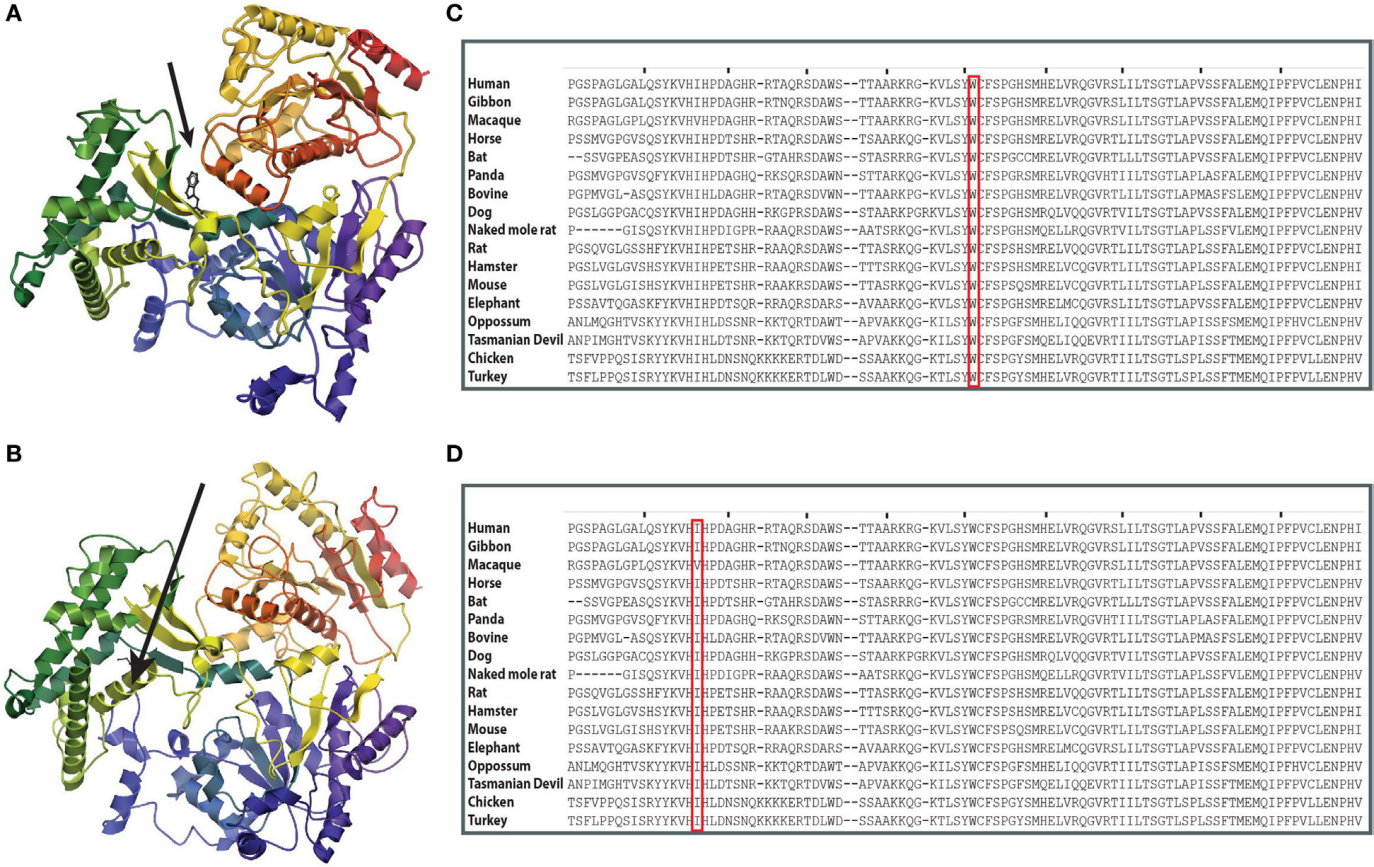
**Structure prediction and evolutionary conservation of *RTEL1* mutations: p.Trp456Cys; p.Ile425Thr. (A)** The mutated aa (p.Trp456Cys) is predicted to be located in a β-strand in the linker between the two helicase domains (arrow) and is predicted to be buried. **(B)** The mutated aa (p.Ile425Thr) is predicted to be located in α-helix. Structure of RTEL1 amino acids (aa) 1 (blue) to 754 (red) was predicted by I-TASSER covering the two helicase domains and their linker based on the structure of 2vsfA (5). Secondary structures were assigned with the program ICM-Browser, Molsoft LLC. **(C)** Trp456 and **(D)** Ile425 are highly conserved across 17 species.

At the time when our index patients P1 and P2 were studied, disease-causing mutations in *RTEL1* were not yet reported. We therefore used Sanger sequencing to exclude mutations in nine known DC-related genes, followed by homozygosity mapping and ES. Three variants (*COL9A3*^Arg103Gln^, *PRIC285*^Glu615Lys^, and *RTEL1*^Trp456Cys^) segregated with the disease (Table 3). *RTEL1* was the most plausible candidate gene, given its essential role in telomere maintenance and coinciding reports on *RTEL1* mutations (Figure 2B). The homozygous mutation p.Trp456Cys affects a protein region with a very high degree of homology in all 17 species and is located in a β-sheet (Figures 3A,C). Targeted re-sequencing identified four additional *RTEL1* mutations in P3-P6 (Figures 2A and 3B). Parents of twins P3 and P4 are first-degree cousins and transmit the heterozygous missense mutation p.Ile425Thr; both P3 and P4 are homozygous, while their healthy siblings are heterozygous carriers (Figure 2A) and have normal RTL (not shown). The RTEL1 amino acid Ile425 is located in the α-helix and found to be conserved in 16 out of 17 tested species (Figures 3B,D). Finally, a compound heterozygous state with one truncating splice site mutation c.2652 + 5G>A in intron 28 and a frameshift mutation c.3730delTG; p.Cys1244ProfsX17 in exon 34b of *RTEL1* was identified in P5 (Figure 2A). P6 was the only patient in this study who carried solely a heterozygous nonsense mutation p.Val796AlafsX4 resulting in premature stop codon prior to both C-terminal harmonin domains. This mutation could not be investigated on functional level in primary patient cells due to lack of available material.

**Table 3:** **Characteristics of the segregating variants in P1 and P2**.

Gene	Position	Ref.	Obs.	Protein	Polyphen-2	SIFT	CADD	ExAC allele counts	ExAC *Z*-score	ExAC pLI
*COL9A3*	chr20 61451333 (rs142639450)	G	A	NM_001853 c.G308A p.R103Q	0.022	0.31	23.5	Het: 1748, hom: 24 (119804); MAF: 0.01459	−1.09	0
*HELZ2*	chr20 62196625	G	A	NM_033405 c.G1843 p.E615K	0.809	0.84	0.002	Het: 78, hom: 1 (70806); MAF: 0.0011	0.25	0
*RTEL1*	chr20 62319010	G	T	NM_016434 c.G1368T p.W456C	1.000	0	28.8	Not observed	−1.71	0.78

*Prediction scores were calculated with Polyphen-2 (42), SIFT (43), and CADD (44). Variant counts (in brackets: total analyzed alleles) and frequencies were downloaded from ExAC database (45) (accession date 21 February 2017) for the whole population*.

*Ref., reference; Obs., observed; het, heterozygous; hom, homozygous; pLI, probability of loss of function intolerance*.

### RTEL1 Isoforms and Consequences of Mutations

**Figure 4:**
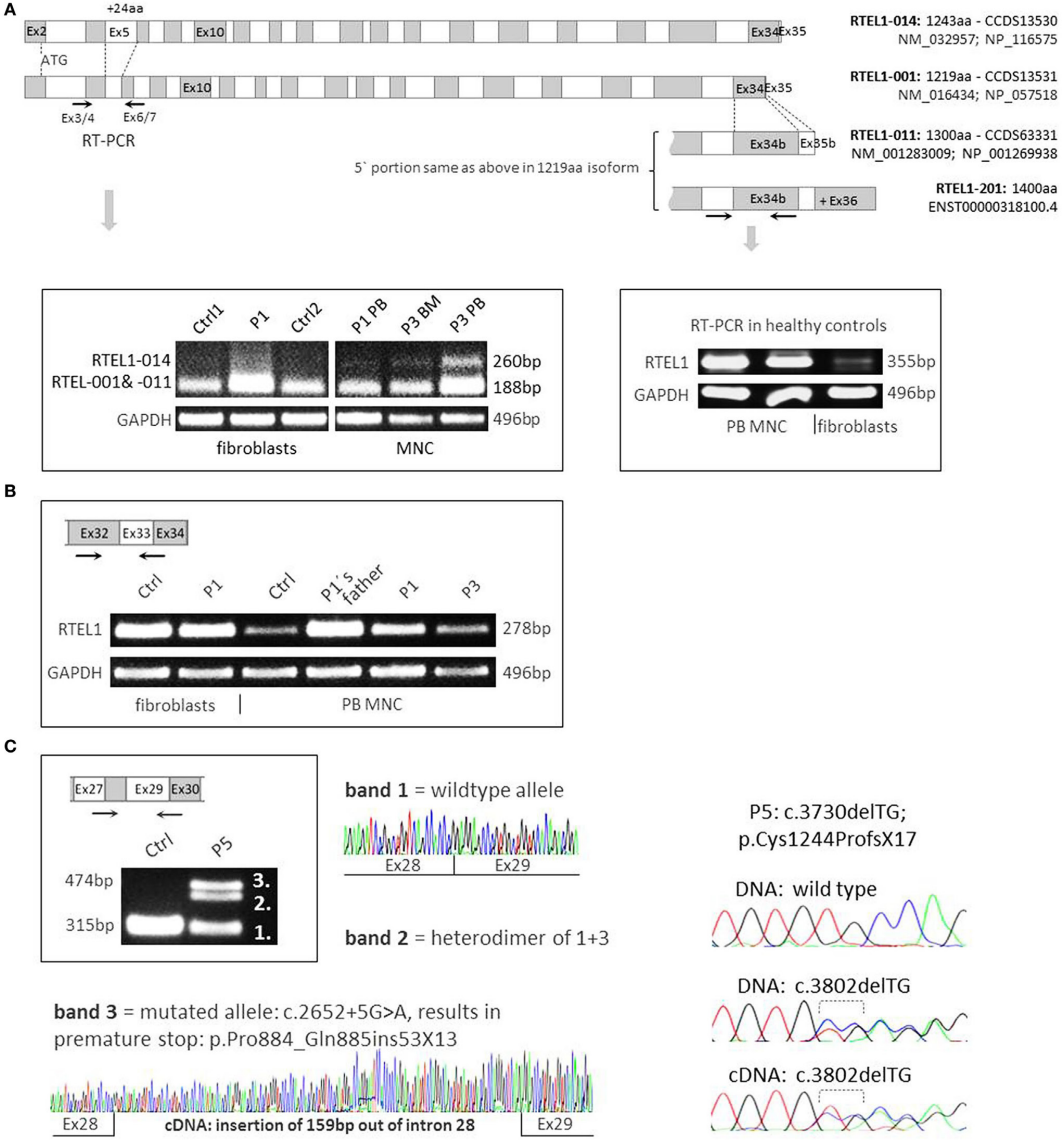
**RNA expression analysis and *RTEL1* isoforms**. **(A)** Four protein coding isoforms are shown and enumerated according to Ensembl nomenclature (RTEL1-014, -001, -011, -201). The RTEL1-014 mRNA isoform containing long exon 5 is only marginally expressed in mononuclear cells (MNCs) from peripheral blood (PB) or bone marrow (BM) and is not detectable in fibroblasts, as shown on the left panel. Right panel depicts higher levels of mRNA containing exon 34b (present in both 1300aa/1400aa isoforms) in control MNC as compared to normal fibroblasts. **(B)** *RTEL1* mRNA expression in fibroblasts, PB MNC of healthy control (Ctrl), P1, P3, and healthy carrier. **(C)** Left panel: mutation c.2652 + 5G>A in P5 was confirmed by RT-PCR to abrogate the original donor splice site, adding +159 bp downstream in intron 28, resulting in an insertion of 53 triplets and a novel stop codon (+13aa). Right panel: second mutation in P5, c.3730delTG is detected in both BM genomic DNA and cDNA at comparable mutant-to-wild-type ratios.

The longest four of the seven Ensembl-annotated, protein coding isoforms are shown in Figure 4A. Mutation nomenclature in this manuscript refers to the 1300aa isoform (NM_001283009), which along with the 1219aa isoform (NM_016434) is predominantly expressed in human cells, and also served as a reference for the majority of reported mutations (Figure 2B). Notably, the 1243aa isoform (NM_032957) that compared to other isoforms holds a longer exon5 (+24aa) was detected at very low levels using RT-PCR in peripheral blood MNCs of patients and controls, while it was absent in fibroblasts, pointing toward tissue-dependent specificity of alternative transcripts (Figure 4A). Furthermore, we noticed higher expression of long transcript (present in both 1300aa/1400aa isoforms) in healthy MNC as compared to fibroblasts (Figure 4B). Finally, the longest 1400aa transcript was neither detectable in blood nor in fibroblasts using RT-PCR targeting exon 36 (not shown).

Both homozygous missense mutations identified in P1–P4 did not affect mRNA expression in RTEL1-deficient patient cells (Figure 4B). As demonstrated by RT-PCR and cDNA sequencing, P5’s *RTEL1* c.2652 + 5G>A mutation abolishes the original donor splice site, resulting in transcription prolongation of exon 28, adding 159 bp out of intron 28 (Figure 4C, left panel). A novel premature stop codon (aa +13) results in loss of the proliferating cell nuclear antigen-interacting protein PIP motif, RING domain, as well as recently identified harmonin-like domains (46). The mutated transcript showed similar mRNA expression signal to the wild-type allele. The second frameshift mutation in P5 was predicted to be protein truncating however did not affect mutant mRNA level as shown by cDNA sequencing (Figure 4C, right panel).

### Severe Telomere Shortening and Premature Senescence in RTEL1 Deficiency

**Figure 5:**
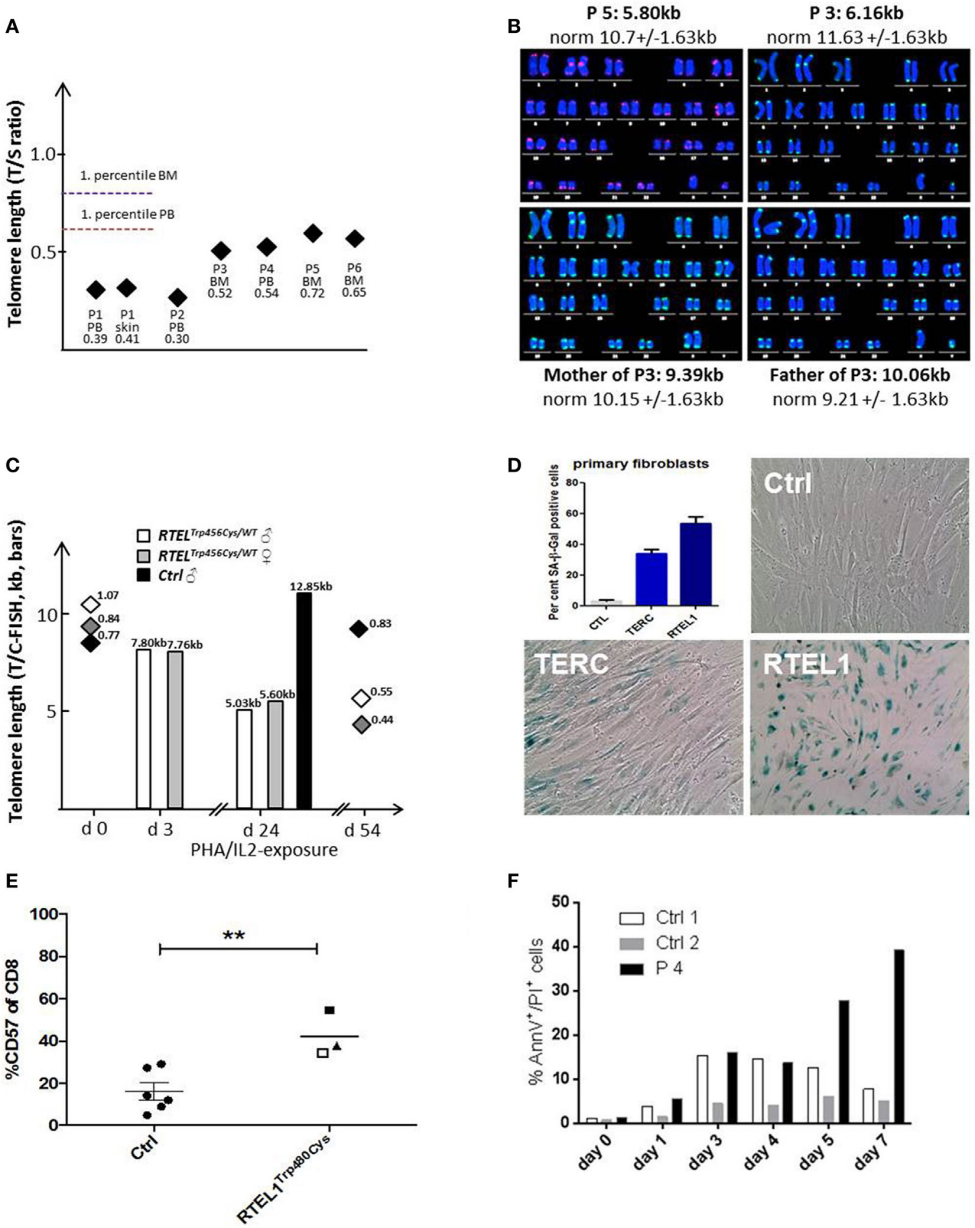
**Telomere shortening, senescence, and spontaneous apoptosis**. **(A)** Relative telomere length (RTL) was measured using qPCR below the first percentile of healthy pediatric population (*n* = 90, age 2–18 years) in Peripheral blood, bone marrow and skin fibroblasts of patients P1–P6. Median of at least two independent triplicate measurements is shown. **(B)** Telomere/centromere metaphase FISH (T/C-FISH) confirms short telomeres in P5 and in P3 but not in heterozygous parents of P3/4. **(C)** Telomere shortening in Trp456Cys-heterozygous T-cells of parents of P1 after PHA/IL2-induced long-term expansion. Bars indicate absolute telomere length examined by T/C-FISH at days 3 and 24 of culture. In addition, RTL h (T/S-ratio by qPCR, diamonds) was investigated at days 0 and 54, confirming rapid telomere shortening observed using T/C-FISH results. **(D)** High rate of senescence visualized by β-galactosidase staining (blue) in RTEL1-deficient fibroblasts, compared to a healthy control and a patient with *TERC* mutation. The percentage of SA-β-Gal positive senescent cells is shown in the bar graph. **(E)** CD57 expression on CD8 cells of age-matched healthy controls (Ctrl) and RTEL1-deficient P3 at 4 years (open square) and 5 years of age (solid square) and P4 at 5 years of age (triangle). **P value <0.01, calculated with a two-tailed *t*-test. **(F)** Increased spontaneous apoptosis in RTEL1-deficient peripheral blood mononuclear cell (PBMNC) of P4 after prolonged crude cell culture. Similar results were observed for P3 PBMNC (not shown).

Patients with RTEL1 deficiency exhibited very short telomeres (below the first percentile of age-matched controls) in BM, PB, and skin fibroblasts measured using qPCR and metaphase T/C-FISH (Figures 5A,B). By contrast, parents of P1–P4 had normal telomere length (Figure 5B). To investigate if heterozygous carrier status affects telomere homeostasis under replicative stress, we investigated T-cell blasts of P1’s parents. Upon long-term expansion, heterozygous RTEL1^Trp456Cys^ lymphocytes exhibited significant telomere shortening (Figure 5C), while homozygous RTEL1^Trp456Cys^ cells died prematurely (not shown). RTEL1-deficient fibroblasts of P1 and P3 showed poor growth and premature cellular senescence (Figure 5D). T-cell phenotyping in P3 and P4 revealed that the majority of CD4 T-cells had a naïve phenotype, while almost 50% of CD8 T-cells were terminally differentiated effectors with senescent phenotype (Figure 5E), as determined by CD57 co-expression (47, 48). The proportion of CD57^+^ T-cells increased over time in P3, despite absence of severe viral infections. Moreover, increased spontaneous apoptosis was observed in MNCs of P3 and P4 (Figure 5F). However, short-term proliferation, degranulation and effector cytokine production of T-cells were unaffected (Table 2).

### Homozygous *RTEL1* Mutations Result in Genomic Instability

**Figure 6:**
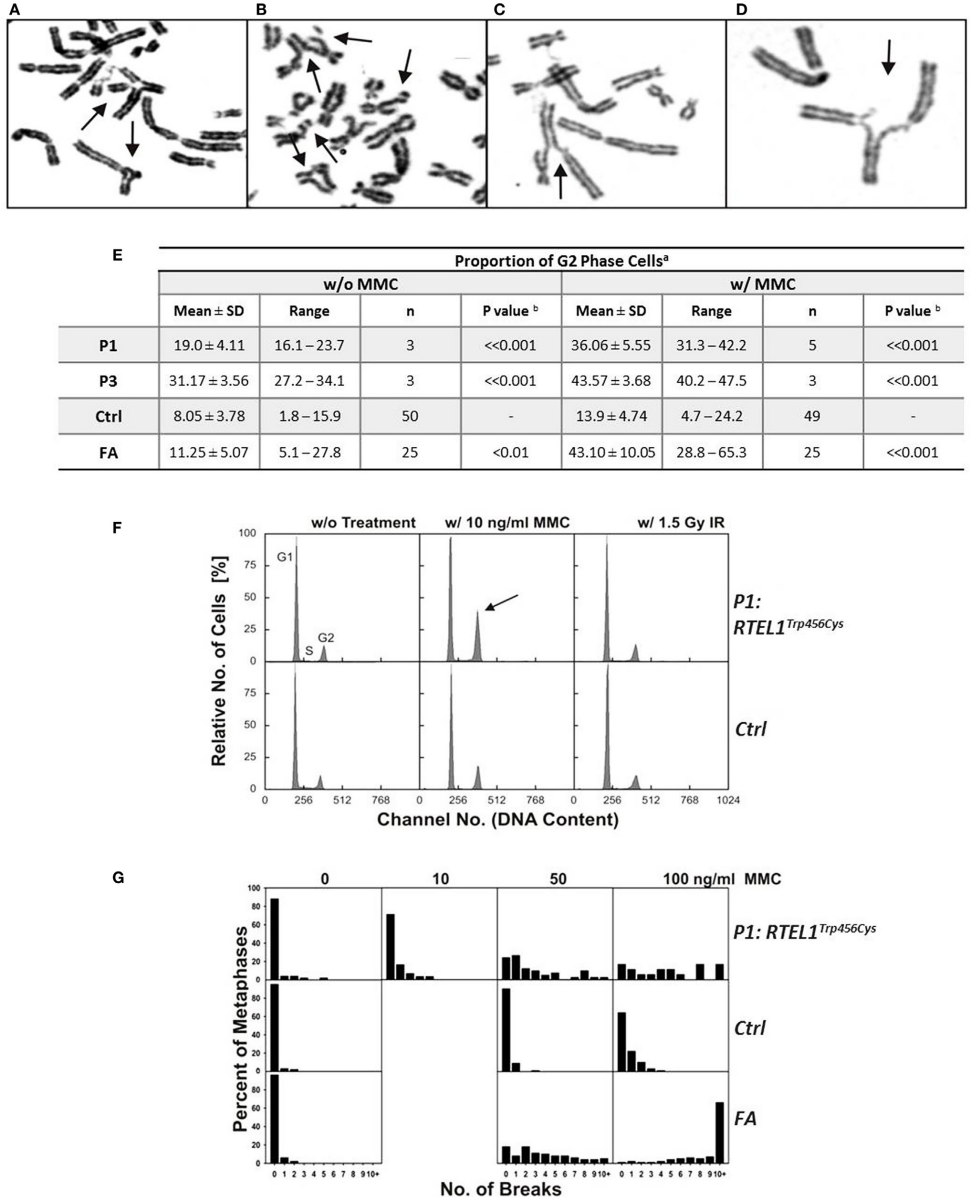
**Spontaneous chromosomal breakage and crosslink induced genomic instability**. **(A–D)** Chromosomal aberrations in P1 fibroblasts exposed to 50 ng/ml mitomycin C (MMC), as indicated by arrows: **(A)** a multiradial and a triradial figure; **(B)** two atypical reunion figures as well as chromatid and chromosome breaks; **(C,D)** radial figures with chromatid breaks. **(E)** Spontaneous and MMC-induced G2 phase accumulation of RTEL1-deficient fibroblasts. Fibroblasts from healthy control (Ctrl) of Fanconi anemia patient (FA) run as controls. ^a^DAPI stained cells from 48 h cultures w/o or w/10 ng/ml MMC; ^b^one-tailed Student’s *t*-test, P1 or P3 compared with normal controls. **(F)** G2 phase accumulation of RTEL1-deficient fibroblasts of P1, DAPI staining. Compared to an untreated healthy control (G1 phase 72.5%, S phase 15.9%, G2 phase 11.6%), fibroblasts from P1 spontaneously show an elevated G2 phase proportion (G1 phase 72.8%, S phase 11.1%, G2 phase 16.1%; left panels). Exposure to 10 ng/ml MMC increases G2 phase in P1 disproportionately (G1 phase 57.4%, S phase 9.5%, G2 phase 33.1%, arrow) compared to a control (G1 phase 69.6%, S phase 7.5%, G2 phase 22.9%). By contrast, 1.5 Gy irradiation reveals comparable G2 phase fractions (P1: 16.0%; control: 20.2%; right panels; DAPI staining). **(G)** Chromosomal break distributions of RTEL1-deficient fibroblasts of P1. Fibroblasts were left untreated or exposed to MMC for the last 24 h. Breakage rates amounted to 0.28 (0 MMC), 0.52 (10 ng/ml MMC), 2.71 (50 ng/ml MMC) or 4.72 (100 ng/ml MMC), respectively. These were elevated compared to normal control fibroblasts with breakage rates of 0.05 (0 MMC; normal mean, 0.02), 0.12 (50 ng/ml MMC; normal mean, 0.18), or 0.55 (100 ng/ml MMC; normal mean, 0.36); the normal mean rate for 10 ng/ml is 0.06. Breakage rates of fibroblast P1 were more evenly distributed among all break classes and did not show the tendency toward metaphases with 10 or more breaks of FA fibroblasts at 100 ng/ml MMC.

Experimental evidence from studying RTEL1-deficient embryonic stem cells, *C. elegans* and human cell lines indicate that RTEL1 is required for maintaining genomic integrity and plays a key role in regulating homologous recombination (HR) (25–27). It is essential for the repair of mitotic and meiotic double strand breaks and its loss results in uncontrolled HR leading to chromosomal breakage. To test the functional effect of *RTEL1* mutations on the genomic integrity in our patients, we investigated spontaneous and crosslinker-induced chromosomal damage (Figure 6). Chromosome preparations from fibroblasts of P1 showed elevated levels of chromatid/iso-chromatid rather than chromosome breaks and reunion figures (Figures 6A,D). End-to-end chromosome fusions were not observed. The spontaneous and MMC-induced G2 phase fractions of fibroblasts from P1 and P3 were significantly increased compared to normal controls (*P* < 0.001) (Figure 6E). By contrast, low dose of ionizing radiation (1.5 Gy) did not result in any abnormal findings (Figure 6F). Without treatment, we observed metaphases with three and five breaks, rarely ever seen in normal controls (Figure 6G). Unexpectedly, repeated chromosomal breakage analysis performed on hematopoietic cells from our patients yielded normal results (Table 1).

### Impaired Proliferative Capacity of B-Cell Precursors and BM Derived CD34^+^ Cells

**Figure 7:**
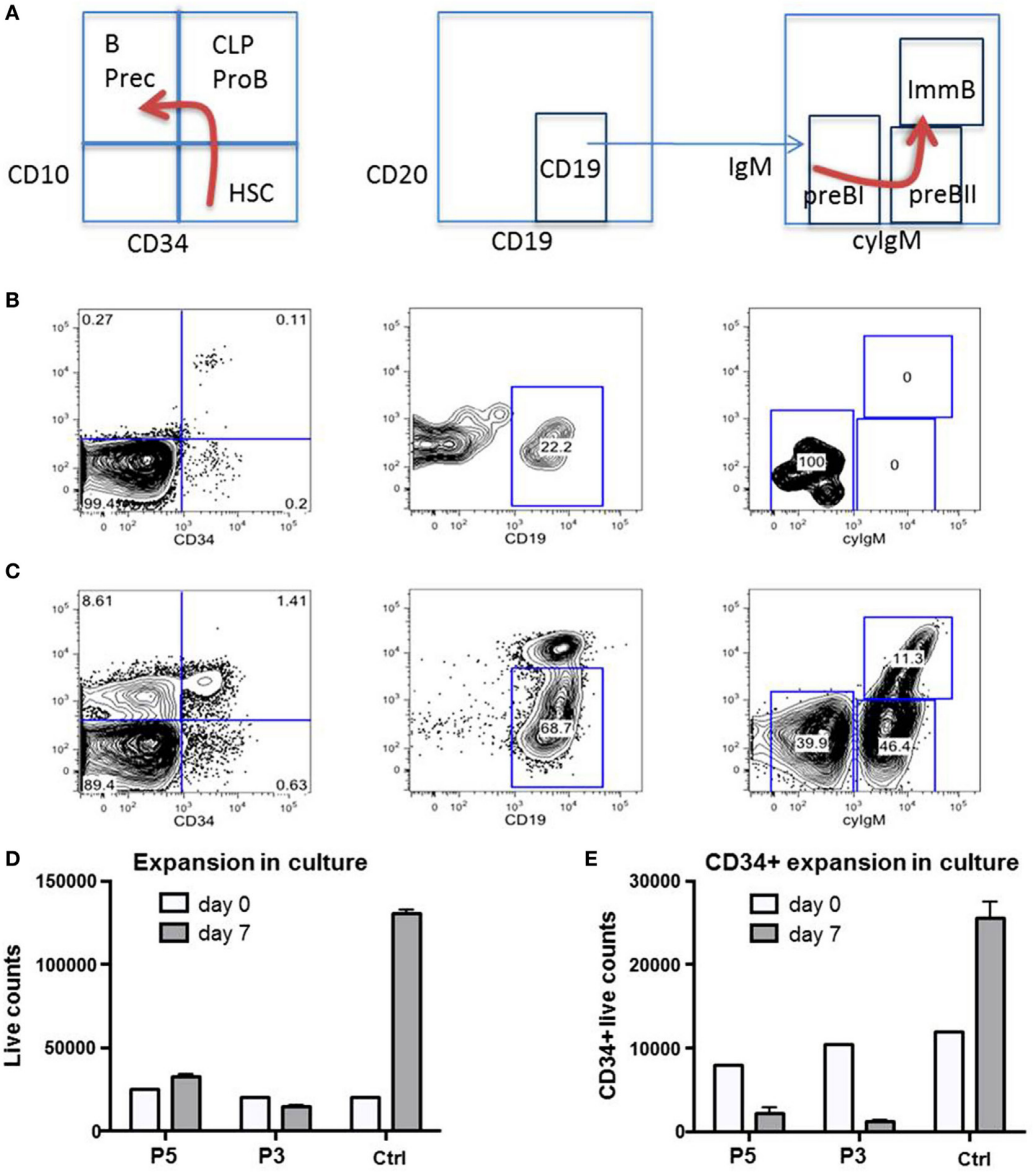
**Impaired proliferative capacity of B-cell progenitors *in vivo* and CD34^+^ cells *in vitro***. **(A)** Normal ontogeny of B-cells in bone marrow (BM). B Prec, B-cell precursors; CLP, common lymphoid progenitor; HSC, hematopoietic stem cells; ImmB, Immature B-cells. **(B)** B-cell differentiation is markedly impaired in BM of P1 during severe adenovirus infection. CLP/ProB subsets are greatly reduced while B cell precursors are missing. **(C)** Between infectious intervals, all populations recover. **(D,E)** Total and CD34^+^ selected HSC from P3 and P5 fail to expand in non-differentiating culture. Ctrl, healthy control.

We had the opportunity to investigate the B-cell distribution in BM and blood of P1 at age 22 months during an acute adenovirus infection and after clearance 3 months later (Figures 7A–C). During acute infection, we observed a differentiation arrest at the level of pre-BI-progenitors, resulting in the absence of pre-BII-progenitors and immature B-cells; and correspondingly very low numbers of peripheral B-cells (Figure 7B). After clearance of infection all previously diminished B-cell populations normalized (Figure 7C). As the cellular immunity relies on continuous replenishment from hematopoietic stem cells (HSC), we additionally investigated the proliferative potential of primary RTEL1-deficient BM cells. While total BM and CD34^+^ cells of P3 and P5 failed to expand *in vitro*, after prolonged culture the CD34^+^ population nearly disappeared (Figures 7D,E).

### Unaffected V(D)J Recombination and T-Circle Accumulation in RTEL1 Deficiency

**Figure 8:**
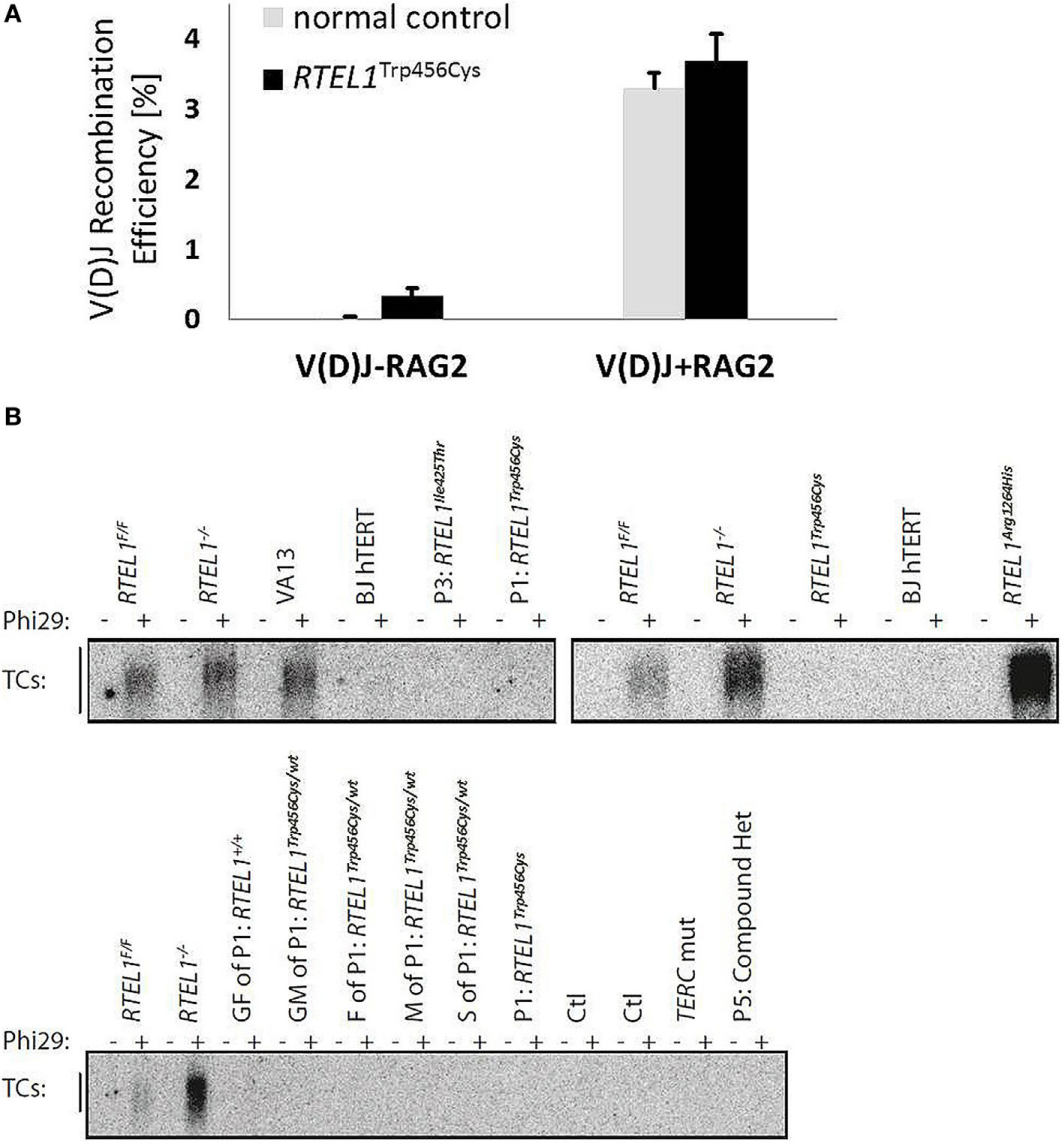
**Unaffected V(D)J recombination and T-circle formation**. **(A)** V(D)J recombination rate in RTEL1-deficient fibroblasts of P1 is comparable to a healthy control. V(D)J-RAG2, RAG1 vector alone; V(D)J + RAG2, RAG1 and RAG2 vectors combined. **(B)** Phi29-dependent telomeric circle (T-circle) amplification assay. Top left: fibroblasts of P1 (p.Trp456Cys) and P3 (p.Ile425Thr) as compared to VA13 ALT cells (a human lung fibroblast cell line that maintains telomeres in the absence of telomerase and accumulates free TC). RTEL1-deficient mouse embryonic fibroblasts (MEFs) were used as positive controls and *Rtel1^−/−^* floxed (F/F) MEFs, and BJhTERT (hTERT immortalized human foreskin fibroblast cell line) as negative controls. Top right: TC accumulation in cells of a patient with a homozygous *RTEL1* mutation p.Arg1264His (previously reported) in comparison to undetectable TC in P1 (p.Trp456Cys). Bottom: TC amplification assay in T-lymphoblasts of P1 and family members (GF, grandfather; GM, grandmother; F, father; M, mother; S, sister), healthy controls (Ctl), a patient with confirmed mutation in *TERC*, and P5 with a compound heterozygous *RTEL1* mutation. T-lymphoblasts were expanded from total peripheral blood mononuclear cell using PHA (lanes 5–18) or anti-CD3/28 beads (lanes 19–24). Phi29, Phi29 DNA polymerase; *Rtel1^F/F^, Rtel1* floxed MEFs before Cre-mediated excision of the *Flox* alleles; *Rtel1^−/−^, Rtel1*-deficient MEFs after Cre-mediated excision of the *Flox* alleles. Wt, wild type.

The progressive B-cell lymphopenia is a frequent observation in the context of impaired somatic recombination, thus we also examined V(D)J recombination in RTEL1-deficient fibroblasts of P1. As depicted in Figure 8A, V(D)J recombination efficiency was unaffected and comparable to healthy controls. Based on recently published experimental data, RTEL1 has been proposed to dismantle T-loops during replication thus preventing catastrophic cleavage of telomeres as a whole extra-chromosomal T-circle (27). Surprisingly, we did not detect elevated T-circle formation in any of our patients or carriers (in fibroblasts, BM and PB) using rolling circle amplification assay when compared to *RTEL1* null mouse cells and RTEL1-deficient human cells carrying the homozygous *RTEL1*^Arg1264His^-mutation (Figure 8B), thus suggesting impaired T-loop disassembly may not be the only underlying cause of the disease.

### HSC Transplantation Corrects the Immunological and Hematological Phenotype of RTEL1 Deficiency

Allogeneic HSCT was performed on P1 and P3 at 3.1 and 5.2 years, respectively. Both patients received non-manipulated BM after reduced intensity conditioning with fludarabine, thiotepa, and antithymocyte globulin. While P3 received BM from a 9/10 MUD, P1 was transplanted from the 48 years old HLA-identical grandmother who was healthy and had RTL within normal range. At HSCT, RTEL1 deficiency was not yet identified in the family and we were unaware of the donor’s heterozygous state for the *RTEL1*^Trp456Cys^-mutation. Full donor chimerism was achieved from day +22 onward and remained stable. At last follow-up (day +1,530), P1 showed persisting hematological and immunological reconstitution. There were no signs of graft-versus-host-disease or chemotherapy-related toxicity. Chronic diarrhea improved with clearance of viral infections. Nonetheless, her esophageal stenosis persists over time and requires frequent dilatation to allow normal oral nutrition. Similar to other recently described DC patients P1 also developed multiple pulmonary arteriovenous malformations with need for continuous oxygen support (20). The course of HSCT in P3 was uneventful. Full donor chimerism was achieved from day +30 onward. At last follow-up at 2 years 6 months post HSCT the patient was 7 years 8 months old, showed good developmental progress and normal hematological and immunological function. His oral leukoplakia showed moderate progress but he did not show signs of esophageal stricture or other mucosal problems so far.

## Discussion

Dyskeratosis congenita represents a genetically heterogeneous group of disorders characterized by telomere shortening leading to BMF and mucocutaneous symptoms (1). Patients with HHS phenotype additionally suffer from cerebellar hypoplasia and immunodeficiency resulting in profound susceptibility for early-onset systemic infections (3, 5). The latter can often be difficult to distinguish from primary defects of lymphocyte development such as severe combined immunodeficiencies (SCID) (8, 49). Immunophenotyping revealed a T^+^B^−^NK^−^ phenotype in P1–P5, which in the context of pancytopenia pointed toward an inherited BMF syndrome. The normal percentage of circulating naïve T-cells suggested a regular thymic output in our patients. Furthermore, normal V(D)J recombination and lack of radiosensitivity argued against an initially assumed B^−^NK^−^ SCID in index patient P1. ES of the index family identified homozygous mutation in the DNA-helicase gene *RTEL1* as the molecular cause of disease. Biallelic *RTEL1* mutations had been identified as the molecular basis in a subset of patients with HHS (9–13). Overall, we found two novel homozygous missense mutations in P1–P4 and three novel truncating mutations in P5 and P6. Homozygous mutations in our patients correlated with onset of symptoms within the first 3 years of life and faster disease dynamics. By contrast, in P5 with biallelic truncating mutations, relevant hematologic problems developed in the second decade of life and were apparently precipitated by an EBV infection. Finally, P6 with only one truncating mutation was identified following a work-up for thrombocytopenia and macrocytosis seen in a routine blood count prior to orthopedic surgery at 17 years of age.

By comparison, the reported mutational landscape for RTEL1 deficiency shows a tendency of clustering at helicase domain 2 and between/at harmonin 1 and 2 (Figure 2B). These mutations, identified in 24 pedigrees with DC are mostly compound heterozygous and in the majority of those cases there is at least one nonsense truncating mutation per case (similar to P5). The rare exception are homozygous *RTEL1*^Phe964Leu^ and *RTEL1*^Arg1264His^-mutations located at C-terminus (10, 13, 14). By contrast, homozygous *RTEL1*^Ile425Thr^ and *RTEL1*^Trp456Cys^-mutations reported here reside closer to N-terminus, in between two functional helicase domains.

Based on the results from cellular and animal models, RTEL1 is essential for DNA replication, and plays a key role in HR and telomere maintenance. In agreement with recent reports, we here show that very short telomeres in various primary tissues, and chromosomal breakage in fibroblasts represent the cellular phenotype of human RTEL1 deficiency (Table 4). It remains to be answered why the genomic instability is restricted to fibroblasts but not hematopoietic cells of RTEL1-deficient patients. One potential explanation might be the tissue-dependent expression of various *RTEL1* isoforms with their specific functionality. Supporting this, we show that different isoforms are expressed at uneven levels in fibroblasts and hematopoietic cells. Our experimental findings on genomic instability reflect a DNA repair disorder of inter-strand crosslinks. The spontaneous G2 phase accumulation and chromosomal aberrations, and pronounced MMC-induced G2 phase arrest of RTEL1-deficient fibroblasts resemble the phenotype of Fanconi anemia fibroblasts of most subtypes. Thus far, genomic instability had not been considered a defining feature in DC, but notably, Dokal and colleagues previously reported spontaneous chromosomal rearrangements in DC-fibroblasts (50). Moreover mouse *Rtel1^−/−^* embryonic stem cells displayed many chromosome breaks and fusions upon differentiation *in vitro* (Table 4) (51). Nevertheless, it is too early to speculate whether, in analogy to genomic instability syndromes, RTEL1 deficiency might be associated with an increased cancer predisposition manifesting later in life. Interestingly, the analysis of mice with deficient RTEL1–PCNA interaction revealed accelerated onset of tumorigenesis in a *p53^−/−^* background (52). However, the clinical histories of all our families and the published data do not support the notion of increased tumor risk. Yet, it is rather difficult to assess cancer predisposition in patients succumbing early in life or when RTEL1 deficiency does not affect global genome integrity. Finally, it is evident that the clinical penetrance is higher and onset much earlier for immunodeficiency, and BMF as opposed to potential tumor susceptibility.

**Table 4:** **Phenotype of RTEL1 deficiency in mouse and human**.

	Mouse	Human
Genetic background and disease severity	*Rtel^−/−^* embryonic lethal	*Compound heterozygous*: DC/HHS (24 patients/18 families) *Homozygous missense*: p.Phe964Leu, p.Arg1264His mutations in DC/HHS (4 patients/3 families), NK cell deficiency (1 patient)^a^
	*Rtel^+/−^* no abnormalities	*Heterozygous nonsense*: hypocellular BMF, DC/HHS^b^ (2 patients/1 family), lung fibrosis (9 patients/6 families) *Heterozygous missense*: thus far only in DC-like disease (1 patient) and pulmonary fibrosis (14 patients/10 families)^c^

Radiosensitivity	No: MEFs *Rtel^−/−^*	No

Crosslinker sensitivity (MMC-induced)	Yes: MEFs *Rtel^−/−^*	Yes: fibroblasts
		No: PB

Chromosomal instability (spontaneous)	Yes: ES *Rtel^−/−^*	Yes: fibroblasts
		No: PB

Telomere shortening	Yes: ES *Rtel^−/−^*	Yes: fibroblasts and PB/bone marrow

BMF and immune deficiency	n.a. (lethal)	Yes: BMF and T^+^B^−^NK^−^ phenotype

Cancer predisposition	n.a. (lethal)	Unknown, so far not reported^d^

*MEFs, mouse embryonic fibroblasts; ES, embryonic stem cells; MMC, mitomycin C; n.a., data not available; +/−, heterozygous; −/−, homozygous; DC, dyskeratosis congenita; HHS, Hoyeraal-Hreidarson syndrome; BMF, bone marrow failure; PB, peripheral blood*.

*^a^Recently, homozygous missense mutation, p.Arg1264His (14) was reported in a 23-month-old girl born with isolated natural killer cell deficiency*.

*^b^So far, only one heterozygous nonsense mutation p.Arg986X (9) was reported in two brothers with severe DC and their healthy mother, in addition to P6 reported here*.

*^c^Ballew et al. (13) reported a heterozygous mutation p.Ala621Thr (equals p.Ala645Thr in NM_032957 isoform) in a patient with BMF and short telomeres; Deng et al. (12) reported on a lung fibrosis in an otherwise healthy family member with heterozygous mutation (p.Met492Ile). Similarly, Newton et al. (21) and Kannengiesser et al. (22) reported pulmonary fibrosis in 11 patients in 5 families (mutations: p.Pro484Leu, p.Pro647Leu, p.Ser688Cys, p.His1124Pro, p.Tyr49Met, p.Arg213Trp, and p.Phe964Leu); Stuart et al. (23) also demonstrated three patients with pulmonary fibrosis and short telomeres harboring heterozygous missense variants p.Pro484Leu, p.Pro647Leu, and p.His1124Pro*.

*^d^Several studies demonstrated an association between RTEL1 rs2297440, rs6010620, and rs6062299 polymorphisms and the risk of glioma (53)*.

T-loop disassembly during replication has been reported as one of the chief functions of RTEL1 (10). This prevents catastrophic cleavage of telomeres as whole T-circles by the SLX4 complex, facilitating the physiological replication of telomeric repeats. Of note, T-circle accumulation was not observed in primary fibroblasts or T cells of our patients with mutations p.Trp456Cys, p.Ile425Thr, p.Pro884_Gln885ins53X13, and p.Cys1244ProfsX17 unlike in recently reported patients with biallelic *RTEL1* mutations (homozygous p.Arg1264His; compound heterozygous p.Leu710Arg/p.Lys897Glu and p.del398_422/p.Arg957Trp) which exhibited substantial T-circle accumulation (10, 13). However, the results of our study are in line with observations made by Deng et al. who also have not identified increased T-circle formation in patient cells with compound heterozygous mutations p.Met492Ile/p.Arg974X (12). In conclusion, it is possible that mutations abolishing the PIP-boxes and thus the RTEL1-PCNA interaction do not result in T-circle formation, as recently reported for the PIP-mutant mouse (12, 52). The mutations characterized by us possibly do not affect regions necessary for T-loop disassembly and the suppression of telomere loss as a whole circle.

We demonstrate a marked vulnerability of RTEL1-deficient cells to replicative stress *in vivo* and *in vitro*. This is in analogy to findings revealing that RTEL1 is indispensable for replication by associating with the replisome and promoting normal genome replication (52). Systemic infections demand for a high replicative turnover, which is markedly limited in RTEL1 deficiency. This is reflected by the insufficient B-cell lineage proliferation, as well as expansion incapability and premature apoptosis of CD34^+^ cells of our patients. It is expected that RTEL1-deficient HSC lose their self-renewal capacity due to impaired telomere replication and cell proliferation, ultimately resulting in proliferative exhaustion of the HSC compartment and progressive BMF. Consequently, the numbers of B-cell precursors diminish over time, leading to hypogammaglobulinemia and loss of specific antibodies in some of our patients. Overall, the replicative capacity seems to be severely limited even in the absence of infectious replicative stimuli. This notion is supported by the observation of premature senescence in fibroblasts and T-cells, in addition to the increased spontaneous apoptosis of RTEL1-deficient MNCs *in vitro*—a mechanism which may expectedly result in a progressive loss of immune cells *in vivo*. Classic tests to evaluate for T-cell effector functions (i.e., short-term proliferation, degranulation, and effector cytokine production) delivered normal results in RTEL1-deficient patients. However, T-cells of heterozygous carriers demonstrated a significant telomere shortening upon mitogen-induced long-term proliferation while homozygous cells die prematurely. We therefore assume that the observed susceptibility to viral disease in RTEL1 deficiency and likely in other DC-subtypes may be a consequence of T-cell exhaustion upon repeated proliferation stimuli triggered by infections. In addition, loss of effector T-cells due to BMF and/or premature senescence as suggested in adult DC patients (49) may have further contributed to secondary T-cell deficiency in our patients.

Several observations indicate that although heterozygous *RTEL1* mutations can facilitate telomere shortening, their pathogenic effect can be compensated by the functional wild-type allele *in vivo*, as opposed to biallelic mutations which result in severe clinical phenotype. First, the missense heterozygotes described in our study were clinically healthy, had normal telomere length, and did not show signs of anticipation. Missense heterozygous *RTEL1* mutations reported thus far did not result in DC phenotype despite telomere shortening in some of the carriers, with the exception of p.Ala621Thr in one patient with DC-like phenotype (Figure 2B; Table 4) as reported by Ballew et al. (9). Notably, majority of the cases reported with solely heterozygous *RTEL1* mutations are found to be associated with telomeropathy-related lung fibrosis (21) and familial pulmonary fibrosis (22, 23) with no immunologic or hematologic phenotypes (Table 4). Second, favorable HSCT outcome with no signs of graft failure more than 4 years after HSCT in P1, who was transplanted from a heterozygous mutation carrier, suggests that certain missense *RTEL1* mutations in heterozygous state may not affect the HSC capacity. However, given the observation that the HSCT procedure itself results in telomere loss corresponding to telomere aging of roughly 15 years cautious long-term monitoring of engraftment is warranted in P1. It should be noted that transplantation from clinically silent heterozygous family member is generally not recommended in DC (54, 55).

The classical mucocutaneous triad pinpointing toward DC (leukoplakia, dystrophic nails, and reticular pigmentation) may be completely missing or only partially present even years after onset of BMF and immunodeficiency in RTEL1-deficient patients. Determination of telomere length could therefore be considered a first-line diagnostic procedure in early-onset immunodeficiency with T^+^B^−^NK^−^ phenotype, particularly when associated with hypocellular BMF. Initially, BMF may only be transient and/or associated with systemic infections.

Therapeutic options are limited in RTEL1-deficient patients. Although androgens can fairly improve blood counts they do not ameliorate the infectious complications. While HSCT offers a potential cure for all BMF-associated symptoms, it also reveals a decision conflict in DC patients. Non-hematological complications (e.g., mucosal fragility and lung fibrosis) are not accessible and may be even aggravated by the procedure itself. However, HSCT can ultimately improve the clinical outcome and the quality of life and may therefore be considered in patients with life-threatening immunodeficiency and BMF. Whether HSCT prevents or increases the risks for tumorigenesis in RTEL1 deficiency remains unanswered at present. Since chemotherapy-related toxicity is excessive in DC independently of the genetic cause, it is obvious that reduced intensity preparative regimens are warranted. In our patients, genomic instability was observed in fibroblasts but not in hematopoietic cells. Nevertheless, alkylating DNA-crosslinking drugs such as busulfan, platins, nitrosoureas, and nitrogen mustards (i.e., cyclophosphamide, melphalan) might have to be considered with caution. Finally, we observe stable hematopoiesis without signs of replicative exhaustion in P1 after HSCT from a family donor with heterozygous *RTEL1* missense mutation. Nevertheless, given the short follow-up observation of 4 years, and in line with previous reports of unfavorable HSCT outcome in a situation with family donor who is DC mutation carrier, the present data are too limited to support HSCT from silent *RTEL1* mutation carriers.

## Ethics Statement

Written informed consent was obtained prior to inclusion of patients. The studies were approved by the institutional review board of the University Hospital Freiburg: protocol number CCI-282/11, studies EWOG-MDS 98 (#NCT00047268) and 2006 (#NCT00662090). The protocol was approved by the local Ethics Committees (CPMP/ICH/135/95 and 430/16).

## Author Contributions

CS, MWW, SS, and KB designed the study and prepared the manuscript; SS, MR, SH, NS, J-BV, GG, KT, ND, UP, AR-E, EkL, and CJ performed experiments; AK, MH, JA, HH, WW, GE, EcL, LR, and BS cared for patients and contributed the clinical data; SB designed and coordinated the T-loop studies and gave conceptual advice; MS performed histological studies; KS designed the V(D)J studies and analyzed data; DS designed the chromosomal breakage studies and analyzed data; SE and CN gave conceptual advice and participated in data analyses, and manuscript writing.

## Conflict of Interest Statement

The authors declare that the research was conducted in the absence of any commercial or financial relationships that could be construed as a potential conflict of interest. The handling editor declared a past co-authorship with the authors and states that the process nevertheless met the standards of a fair and objective review.
