# Corrigendum: Clinical and Molecular Heterogeneity of RTEL1 Deficiency

**DOI:** 10.3389/fimmu.2017.01250

**Published:** 2017-10-02

**Authors:** Carsten Speckmann, Sushree Sangita Sahoo, Marta Rizzi, Shinsuke Hirabayashi, Axel Karow, Nina Kathrin Serwas, Marc Hoemberg, Natalja Damatova, Detlev Schindler, Jean-Baptiste Vannier, Simon J. Boulton, Ulrich Pannicke, Gudrun Göhring, Kathrin Thomay, J. J. Verdu-Amoros, Holger Hauch, Wilhelm Woessmann, Gabriele Escherich, Eckart Laack, Liliana Rindle, Maximilian Seidl, Anne Rensing-Ehl, Ekkehart Lausch, Christine Jandrasits, Brigitte Strahm, Klaus Schwarz, Stephan R. Ehl, Charlotte Niemeyer, Kaan Boztug, Marcin W. Wlodarski

**Affiliations:** ^1^Faculty of Medicine, Department of Pediatrics and Adolescent Medicine, Division of Pediatric Hematology and Oncology, Medical Centre, University of Freiburg, Freiburg, Germany; ^2^Center for Chronic Immunodeficiency, Medical Center, Faculty of Medicine, University of Freiburg, Freiburg, Germany; ^3^Spemann Graduate School of Biology and Medicine, University of Freiburg, Freiburg, Germany; ^4^Faculty of Biology, University of Freiburg, Freiburg, Germany; ^5^Faculty of Medicine, Department of Rheumatology and Clinical Immunology, Medical Centre, University of Freiburg, Freiburg, Germany; ^6^Department of Pediatrics, University of Bern, Bern, Switzerland; ^7^Ludwig Boltzmann Institute for Rare and Undiagnosed Diseases, Vienna, Austria; ^8^CeMM Research Center for Molecular Medicine of the Austrian Academy of Sciences, Vienna, Austria; ^9^Department of Pediatric Hematology and Oncology, Children’s Hospital, University of Cologne, Cologne, Germany; ^10^Department of Medical Genetics, Biozentrum, University of Wuerzburg, Wuerzburg, Germany; ^11^Telomere Replication and Stability Group, MRC London Institute of Medical Sciences (LMS), London, United Kingdom; ^12^Institute for Transfusion Medicine, Institute for Clinical Transfusion Medicine and Immunogenetics Ulm, German Red Cross Blood Service Baden-Wuerttemberg – Hessen, University Ulm, Ulm, Germany; ^13^Department of Human Genetics, Hannover Medical School, Hannover, Germany; ^14^Department of Pediatric Hematology and Oncology, Justus Liebig-University, Giessen, Germany; ^15^Clinic of Pediatric Hematology and Oncology, University Medical Center Hamburg Eppendorf, Hamburg, Germany; ^16^Hemato-Oncology Clinic Hamburg, Hamburg, Germany; ^17^Faculty of Medicine, Institute of Pathology, Medical Center, University of Freiburg, Freiburg, Germany; ^18^German Cancer Consortium (DKTK), Freiburg, Germany; ^19^German Cancer Research Center (DKFZ), Heidelberg, Germany; ^20^Department of Pediatrics and Adolescent Medicine, Medical University of Vienna, Vienna, Austria; ^21^Department of Pediatrics and Adolescent Medicine, St. Anna Kinderspital and Children’s Cancer Research Institute, Medical University of Vienna, Vienna, Austria

**Keywords:** RTEL1, dyskeratosis congenita, bone marrow failure, immunodeficiency, lymphopenia

Missing Funding

In the original article, we neglected to include the funder **[Excellence Initiative of the German Research Foundation**], **[GSC-4, Spemann Graduate School]** to **[Sushree Sangita Sahoo]**. The authors apologize for this error and state that this does not change the scientific conclusions of the article in any way.

Addition of Affiliation

In the published article, there was an error regarding the affiliation for **[Sushree Sangita Sahoo]**. As well as having Affiliation **[1,3]**, she should also have **[Faculty of Biology, University of Freiburg, Germany]**. The authors apologize for this error and state that this does not change the scientific conclusions of the article in any way.

## Conflict of Interest Statement

The authors declare that the research was conducted in the absence of any commercial or financial relationships that could be construed as a potential conflict of interest.

